# Colon Capsule Endoscopy vs. CT Colonography Following Incomplete Colonoscopy: A Systematic Review with Meta-Analysis

**DOI:** 10.3390/cancers12113367

**Published:** 2020-11-13

**Authors:** Ulrik Deding, Lasse Kaalby, Henrik Bøggild, Eva Plantener, Mie Kruse Wollesen, Morten Kobaek-Larsen, Siri Juul Hansen, Gunnar Baatrup

**Affiliations:** 1 Department of Clinical Research, University of Southern Denmark, 5230 Odense M, Denmark; Lasse.Kaalby.Moller@rsyd.dk (L.K.); Morten.Kobaek.Larsen@rsyd.dk (M.K.-L.); gunnar.baatrup@rsyd.dk (G.B.); 2 Department of Surgery, Odense University Hospital, 5000 Odense C, Denmark; Eva.Marie.Huulvei.Plantener@rsyd.dk (E.P.); mie.kruse.wollesen@rsyd.dk (M.K.W.); Siri.Juul.Hansen@rsyd.dk (S.J.H.); 3 Public Health and Epidemiology Group, Department of Health Science and Technology, Aalborg University, 9220 Aalborg Ø, Denmark; boggild@hst.aau.dk; 4 Unit of Clinical Biostatistics, Aalborg University Hospital, 9000 Aalborg, Denmark

**Keywords:** colon capsule endoscopy, CT colonography, computed tomography colonography, colonoscopy, incomplete investigation, colon cancer, polyps, colorectal cancer, meta-analysis, systematic review

## Abstract

**Simple Summary:**

The quality and completeness of colonoscopies might be hampered by suboptimal visualization; often leading to a CT colonography. Colon capsule endoscopy has been suggested as an alternative to CT colonography following incomplete colonoscopy. Colon capsule endoscopy is conducted by the ingestion of a video capsule, which then transmits pictures, allowing complete visualization of the colon and rectum. In our study, we compared the completion rates and diagnostic yields (detection of polyps) of colon capsule endoscopy with those of CT colonography by conducting a systematic review with meta-analysis. We found that the completion rate of CT colonography was slightly higher than that of colon capsule endoscopy, while the diagnostic yield of colon capsule endoscopy was higher, especially when comparing findings of any size polyps. This knowledge might aid clinicians when planning further diagnostics following incomplete colonoscopies.

**Abstract:**

Following incomplete colonoscopy (IC) patients often undergo computed tomography colonography (CTC), but colon capsule endoscopy (CCE) may be an alternative. We compared the completion rate, sensitivity and diagnostic yield for polyp detection from CCE and CTC following IC. A systematic literature search resulted in twenty-six studies. Extracted data included inter alia, complete/incomplete investigations and polyp findings. Pooled estimates of completion rates of CCE and CTC and complete colonic view rates (CCE reaching the most proximal point of IC) of CCE were calculated. Per patient diagnostic yields of CCE and CTC were calculated stratified by polyp sizes. CCE completion rate and complete colonic view rate were 76% (CI 95% 68–84%) and 90% (CI 95% 83–95%). CTC completion rate was 98% (CI 95% 96–100%). Diagnostic yields of CTC and CCE were 10% (CI 95% 7–15%) and 37% (CI 95% 30–43%) for any size, 13% (CI 95% 9–18%) and 21% (CI 95% 12–32%) for >5-mm and 4% (CI 95% 2–7%) and 9% (CI 95% 3–17%) for >9-mm polyps. No study performed a reference standard follow-up after CCE/CTC in individuals without findings, rendering sensitivity calculations unfeasible. The increased diagnostic yield of CCE could outweigh its slightly lower complete colonic view rate compared to the superior CTC completion rate. Hence, CCE following IC appears feasible for an introduction to clinical practice. Therefore, randomized studies investigating CCE and/or CTC following incomplete colonoscopy with a golden standard reference for the entire population enabling estimates for sensitivity and specificity are needed.

## 1. Introduction

During incomplete colonoscopy (IC), the scope rarely reaches the ascending colon (4–25%), and colonoscopy is often terminated in the sigmoid colon (22–43%) [1,2,3,4] or the rectum (6–10%) [3,5], necessitating further evaluation of the colon. Usually, this is performed by repeat colonoscopy or computed tomography colonography (CTC). European guidelines suggest colon capsule endoscopy (CCE) as another option for this purpose [6]. CTC and CCE can both be used to complement IC results, because the proximal parts of the colon are visualized [5,7,8]. CTC may be more reliable for larger lesions (>9 mm) than smaller [9]. If CCE is better for detecting small lesions compared to CTC, it may still be inhibited by a completion rate that is dependent on the battery lifetime exceeding the total transit time of the capsule. CCE after IC may not need to be complete if it is conclusive (i.e., has a positive finding or reaches the most proximal point seen during the IC) [3,4,10,11]. The overall goal should therefore be that individuals must have a complete colonic visualization following IC and CCE. CTC is a well-established and widely implemented diagnostic method [12], whereas the clinical use of CCE is still limited. The number of studies investigating CCE following IC have also been limited but have increased in recent years [2,5,10,11].

The incompletion of colonoscopy is often ascribed to nonpassable segments, stenosis or other obstacles of passage, which are thought to potentially increase the risk of capsule retention, since the highest rate of capsule retention in small bowel capsule endoscopies has been due to obstructions [13]. Research from our unit has shown that the completion rates of CCE after IC (68%) and CCE in patients in a surveillance program (67%) are comparable. Although, a higher proportion of inadequate bowel cleanliness was seen following IC (22%) compared to patients in a surveillance program with previous colonoscopy (7.8%) [5,14]. The CCE can be administered immediately after the IC [3] (or after supplementary bowel preparation, if necessary) in the same unit, which seems patient-friendly. Furthermore, CCE appear to be more tolerable for patients than CTC [15].

We aimed to investigate whether the quality of CCE is worse, equal to or better than that of CTC after IC. Therefore, the aim of this systematic review with meta-analysis was to compare the completion rate, sensitivity and diagnostic yield for polyps from CCE and CTC after IC.

## 2. Materials and Methods

### 2.1. Search Strategy

A systematic literature search was conducted in databases PubMed, Web of Science and Embase. Three areas of search terms were defined: investigation, indication and study design. Investigation was to identify studies comparing colonoscopy with CCE and/or CTC. Indication was to limit results to references in which participants had undergone IC. Study design was to limit results to retro- or prospective cohorts or clinical studies, paired studies (to identify studies testing CCE and CTC in the same patient group), randomized controlled trials (RCTs) and case-control designs. Search terms within each area were combined in search strings using the Boolean expression “OR”. The three areas were then combined in strings using the Boolean “AND”. Free text search terms with truncation were included, as well as indexed search terms identified in the databases’ thesauruses. The only limitation set was to articles, journal articles and articles in the press (depending on search engine options). No limitation for publication years was applied. The final literature search was conducted September 10, 2020. Specific search strings are provided in Appendix A, with search strategy in Table A1.

### 2.2. Inclusion and Exclusion Criteria

RCTs, paired studies, cohort or case-control studies describing an adult population (≥18 years) where individuals have undergone IC followed by either CTC, CCE or both were included. Studies including exclusively occlusive cancer patients were excluded, as this patient subgroup might not be suitable for CCE diagnostics, rendering any clinical potential obsolete. Included were only articles written in the English language.

### 2.3. Screening of References

References, including abstracts, were exported from each database and imported to Endnote™, version X9 [16]. Duplicates were identified and excluded. Title and abstract of remaining references were screened independently by two of three authors (M.K.W., S.J.H. or U.D.) and were excluded if they did not meet the inclusion criteria. In case of discrepancy between screeners, the reference remained included. Full-text manuscripts of references not excluded were retrieved and thoroughly read by three of the authors (U.D., L.K. and E.P.) independently and were excluded if they did not meet inclusion criteria. In case of discrepancies, the authors would reread the article and discuss it again. If further discrepancy was evident, the first author (U.D.) would make the decision whether or not to include the reference. The reference lists of included studies were screened by at least two screeners (U.D., M.K.W., L.K. or E.P.), and any reference of possible relevance would be retrieved in full-text, examined by three authors (U.D., L.K. and E.P.) and included if inclusion criteria were met, and this process continued until exhaustion (snowballing). Reviewers were not blinded to authors and institutions of the reviewed manuscripts.

### 2.4. Data Handling

From each included study, two individuals (U.D. and L.K.) independently extracted the data needed for statistical analysis (Table 1). Differing interpretations were solved in the same manner as with discrepancies regarding the inclusion of studies (see Section 2.3).

Completion rates were then calculated as proportions of investigations in the studies (I) that were complete (IV (V for a complete colonic view rate in CCE)). Sensitivities were to be calculated as true positives (VI) divided by true positives (VI) and false negatives (IX) summed. Diagnostic yields were calculated as proportions of individuals (I) who had at least one polyp, one polyp >5 mm or one polyp >9 mm (III), respectively. Polyp sizes reported in the studies were assumed to follow the standard of reporting the largest diameter of the lesions.

Additional descriptive data were extracted for subgroup analyses. However, due to an insufficient number of articles within each stratum, these analyses could not be performed. For this reason, only some of these data were used for descriptive reasons. These data included the first author, publication year, data origin (country), year of data collection, study type, visualization instrument(s) (type of scanner or capsule), indications for colonoscopy, single- or multi-center study, reported bowel/procedure preparation medicine (incl. boosters and contrast agents), type of reference standard, whether the study included occlusive cancer patients or patients with stenosis or other obstructions, gender distribution and mean age.

In cases of doubt on the meaning or phrasing in an article, a precautionary principle was applied, and the information would not be included in the analyses. Therefore, an article clearly reporting the number of polyps found but being vague about the sizes would only be included for the analysis of polyps of any size. Furthermore, studies being exact with the number of total polyps but unclear on the distribution of polyps in the population would be excluded from diagnostic yield analyses but could still be included for completion rate analyses. Studies not explicitly stating the number of complete or incomplete investigations were not included for analysis of completion rates.

### 2.5. Pooled Statistical Analysis

The proportion of patients with true polyp detection out of the total number of patients with polyp detection (per patient sensitivity) of CCE and CTC was compared using studies with reference standard follow-ups (second colonoscopy) stratified by polyp sizes. We calculated the completion rates of CTC and CCE, as well as the complete colonic visualization rate of CCE following IC. Significance level was set at 5%, and 95% confidence intervals were calculated. All pooled estimates were calculated from patient data of included studies in random effects models using the Freeman-Tukey double arcsine transformation.

Heterogeneity was tested by performing I^2^ statistics to test the consistency of results and evaluated by applying thresholds provided by the Cochrane Handbook [17]. As no limit was applied to the publication year of included articles, a sensitivity analysis for the diagnostic yield of any size polyps, as well as completion rates, was conducted, including only studies published in the last decade (2010 and later).

Publication bias and small study effects were investigated using Egger’s test [18] and illustrated by funnel plots for completion rate, diagnostic yield and sensitivity. All results from the included studies were extracted and compiled in spreadsheets. All data analyses were conducted using Stata 16 [19] including the Metaprop command [20].

## 3. Results

**Figure 1:**
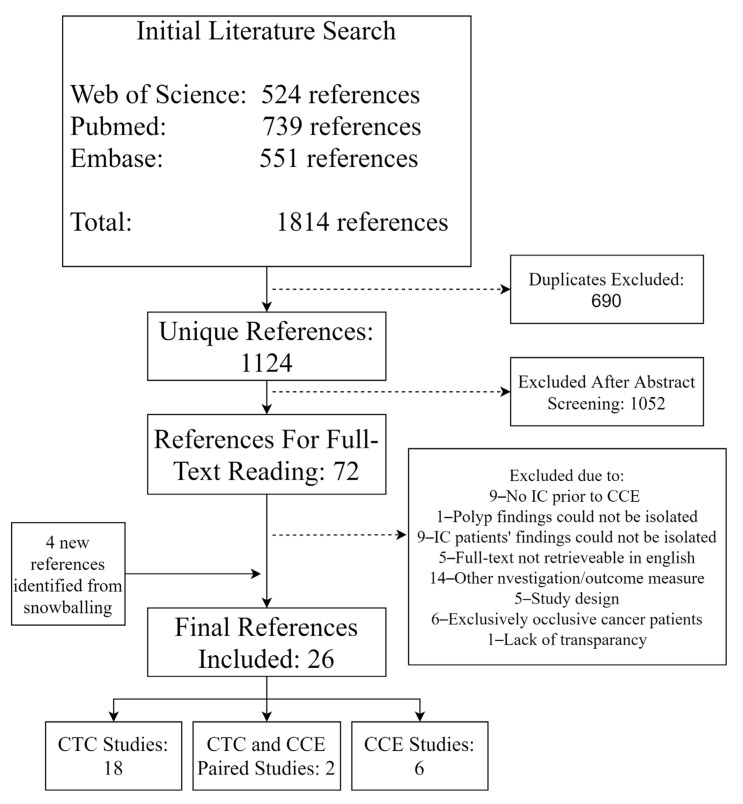
Flow of references from the initial literature search 10th of September 2020 on colon capsule endoscopy vs. CT colonography following incomplete colonoscopy. CTC: computed tomography colonography, CCE: colon capsule endoscopy and IC: incomplete colonoscopy.

The initial literature search resulted in a total of 1814 references. Duplicates were removed (690 references), and 1052 references were excluded after title and abstract screening. After the full-text reading of 72 references, 22 were included in the study. Thorough snowballing yielded a further 25 studies for the screening of their abstracts, of which seven were included for full-text reading. This resulted in a further four included studies (Figure 1). In total, 26 studies were included in this meta-analysis [1,2,3,4,5,10,11,21,22,23,24,25,26,27,28,29,30,31,32,33,34,35,36,37,38,39].

An overview of the 26 included studies is provided in Table 2. Eighteen studies conducted CTC following IC, six studies performed CCE following IC and two studies performed both CCE and CTC following IC in the same group of patients (Figure 1). Therefore, patients from 20 studies were included for CTC analyses and from eight studies for CCE analyses. Only a subgroup of patients had undergone IC previous to CTC in seven studies of CTC, which was also the case in one CCE study (Table 2). In those eight studies, only the subgroup in question was included for analyses. The mean age of individuals in the included studies ranged from 57 to 81 years, with the majority of studies in the range of 60 to 66 years. All but three studies reported the inclusion of more females than males. All CCE studies were from European population samples, whereas CTC studies with European, Asian and American samples were included.

### 3.1. Completion Rate 

**Figure 2:**
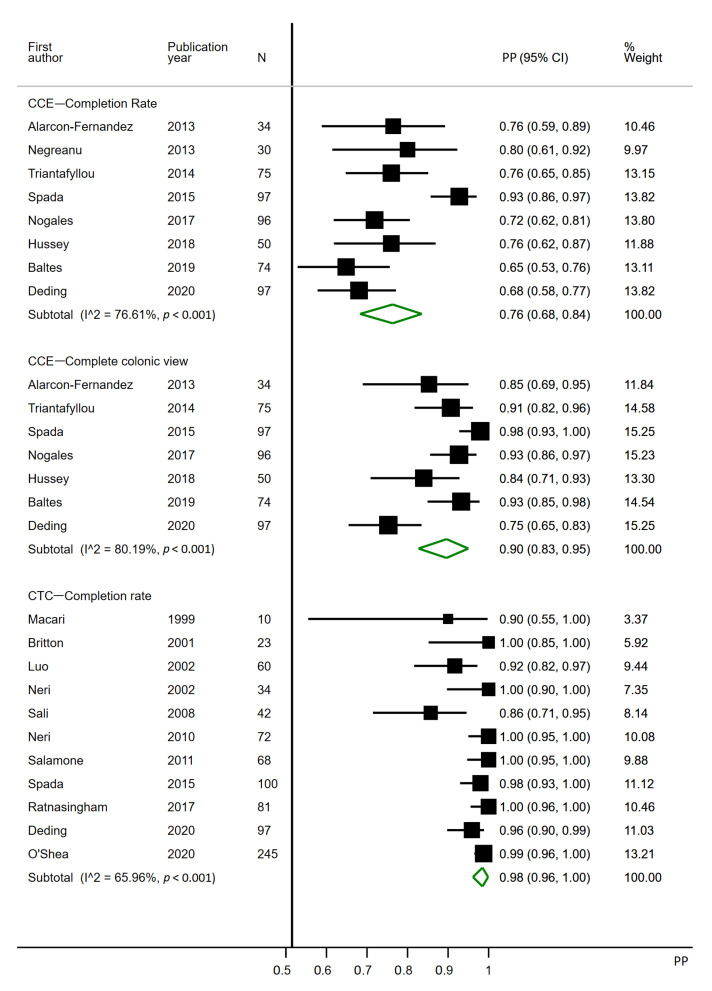
Forest plot for the completion rates of colon capsule endoscopy and CT colonography following incomplete colonoscopy. PP: estimated prevalence proportion. Complete colonic view: CCE investigations complete or overlapping with the most proximal point of a previous incomplete colonoscopy.

All eight CCE studies were included for the completion rate analysis, although one was excluded for complete colonic view [39], as it did not clearly define how this was ensured. Eleven CTC studies were included for the completion rate analysis (Figure 2). Nine CTC studies were excluded from the completion rate analysis, as they did not explicitly report a completion rate or absolute numbers of incomplete/complete investigations [1,21,22,23,24,26,33,36,37].

The completion rate of CCE ranged from 65 to 93%, with a pooled estimate of 0.76 (95% CI 0.68–0.84). The completion rate of CCE, defined as a complete colonic view (CCE reaches the most proximal point of IC), ranged from 75 to 98%, with a pooled estimate of 0.90 (95% CI 0.83–0.95). The completion rate of CTC ranged from 86 to 100%, with a pooled estimate of 0.98 (95% CI 0.96–1.00). Heterogeneities were high in all three subgroups, with I^2^ at 76.61%, 80.19% and 65.96% (Figure 2).

### 3.2. Diagnostic Yield

Diagnostic yields were estimated and reported stratified to polyp sizes: any size, >5 mm and >9 mm.

#### 3.2.1. Polyps Any Size

**Figure 3:**
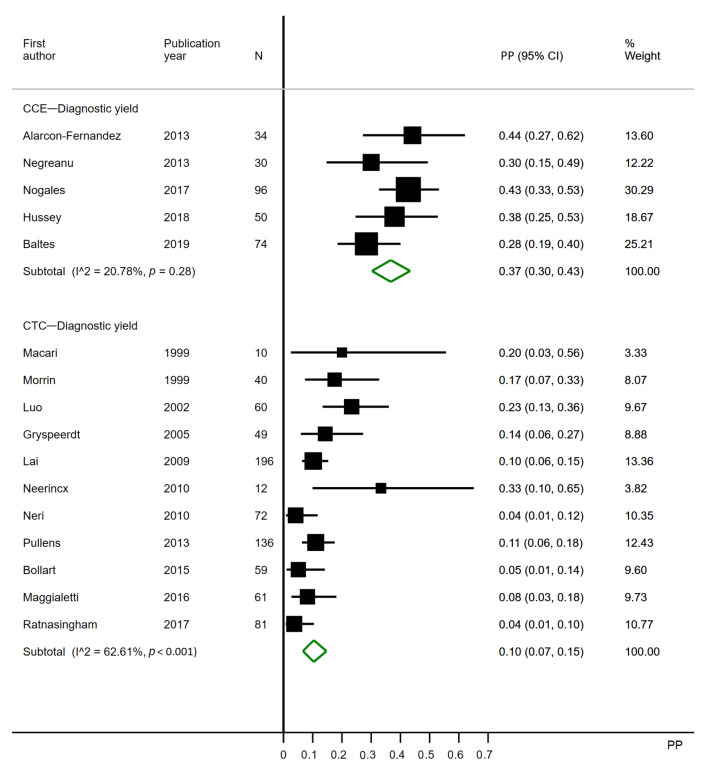
Forest plot of the per patient diagnostic yields for polyps of any size in colon capsule endoscopy and in CT colonography following incomplete colonoscopy. PP: estimated prevalence proportion.

Five CCE studies were included for the analysis (Figure 3). Two CCE studies were excluded, as they did not report polyps <6 mm [4,5], and one study did not report the polyps per patient [3]. Eleven CTC studies were included for the analysis. Three CTC studies were excluded, as they did not report polyps <6 mm [4,5,36]; one study only reported polyps >9 mm [37], and five studies did not report polyps at all [28,29,30,32,38].

The diagnostic yield of CCE ranged from 28% to 44%, with a pooled estimate of 0.37 (95% CI 0.30–0.43). The diagnostic yield of CTC ranged from 4% to 33%, with a pooled estimate of 0.10 (95% CI 0.07–0.15). Heterogeneity may not be present in CCE analyses (I^2^ = 20.78%), which is supported by the *p*-value of 0.28, but is substantial in CTC analyses (I^2^ = 62.61%) (Figure 3).

Limiting the analyses to studies published after 2009 caused no change in relation to the effect for CCE, but in the CTC analysis, five studies were further excluded, and the pooled estimate was reduced to 0.07 (95% CI 0.03–0.12).

#### 3.2.2. Polyps over 5 mm

**Figure 4:**
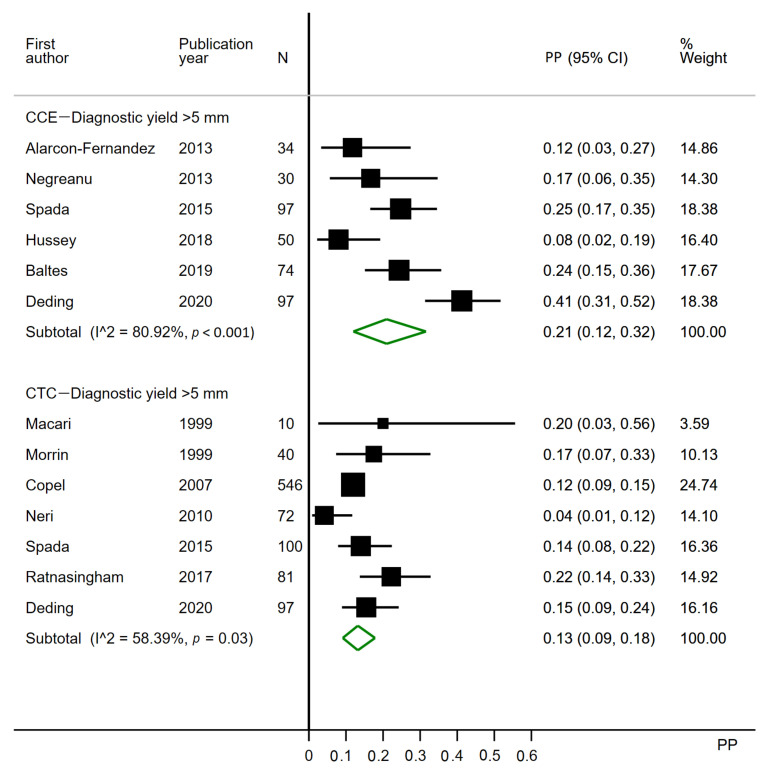
Forest plot of per patient diagnostic yields for polyps >5 mm in colon capsule endoscopy and in CT colonography following incomplete colonoscopy. PP: estimated prevalence proportion.

Six CCE studies were included for analysis (Figure 4). One CCE study was excluded, as it did not report the polyps per patient [3] and one as it grouped patients with polyps >5 mm with those having three or more polyps of any size [2]. Seven CTC studies were included. Five CTC studies were excluded, as they only reported any size polyps [1,21,24,26,34], five studies as they did not report polyps at all [28,29,30,32,38], one as it only reported polyps >9 mm [37], one as it reported polyps any size and polyps >9 mm [22] and one as only polyps >9 mm were reported per patient [33]. Two studies reported polyps >4 mm and not >5 mm but were included anyway [23,25].

The diagnostic yield of CCE ranged from 8% to 41%, with a pooled estimate of 0.21 (95% CI 0.12–0.32). The diagnostic yield of CTC ranged from 4% to 22%, with a pooled estimate of 0.13 (95% CI 0.09–0.18). Heterogeneity was substantial to considerable in the CCE analysis (I^2^ = 80.92%) and moderate to substantial in the CTC analysis (I^2^ = 58.39%) (Figure 4).

Limiting the analyses to studies published post-2009 caused no effect for CCE, but in CTC, analysis three studies were further excluded, and the pooled estimate was unchanged at 0.13 (95% CI 0.07–0.21)

#### 3.2.3. Polyps over 9 mm

**Figure 5:**
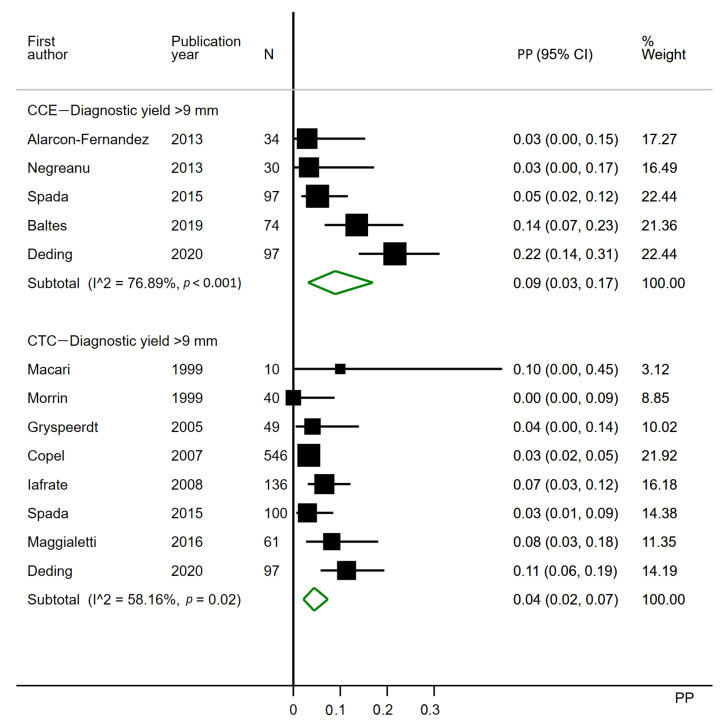
Forest plot of the per patient diagnostic yields for polyps >9 mm in colon capsule endoscopy and in CT colonography following incomplete colonoscopy. PP: estimated prevalence proportion.

Five CCE studies were included for the analysis (Figure 5). One CCE study was excluded, as it did not report polyps per patient [3] and two as polyps >9 mm could not be differentiated from smaller polyps [2,10]. Eight CTC studies were included. Five CTC studies were excluded, as they only reported any size polyps [1,21,24,26,34], two as polyps >9 mm could not be differentiated from smaller polyps [25,27] and five as they did not report polyps at all [28,29,30,32,38].

The diagnostic yield of CCE ranged from 3% to 22%, with a pooled estimate of 0.09 (95% CI 0.03–0.17). The diagnostic yield of CTC ranged from 0% to 11%, with a pooled estimate of 0.04 (95% CI 0.02–0.07). Heterogeneity was substantial to considerable in the CCE analysis (I^2^ = 76.89%) and moderate to substantial in the CTC analysis (I^2^ = 58.16%) (Figure 5).

Limiting the analyses to studies published post-2009 caused no effect for CCE, but in the CTC analysis, four studies were further excluded, and the pooled estimate was increased to 0.07 (95% CI 0.03–0.13).

### 3.3. Sensitivity

None of the included studies performed a reference standard follow-up in all patients. Most of the studies performed colonoscopy or surgery after CCE/CTC investigations showed significant findings. Therefore, pooled sensitivity analyses were omitted. Two studies reported relative sensitivities (the number of positive CCE investigations divided by the number of positive CTC investigations), as they had performed both CCE and CTC in the same sample. The relative sensitivities of CCE compared to CTC were reported to be 2.0 (CI 95% 1.34–2.98) and 2.67 (CI 95% 1.76–4.04) for polyps >5 mm and 1.67 (CI 95% 0.69–4.00) and 1.91 (CI 95% 1.18–3.09) for polyps >9 mm [4,5], indicating a higher sensitivity of CCE compared to CTC. This is a result of CCE finding substantially more polyps compared to CTC in the same groups of patients.

### 3.4. Small Study Effects and Publication Bias

**Figure A1:**
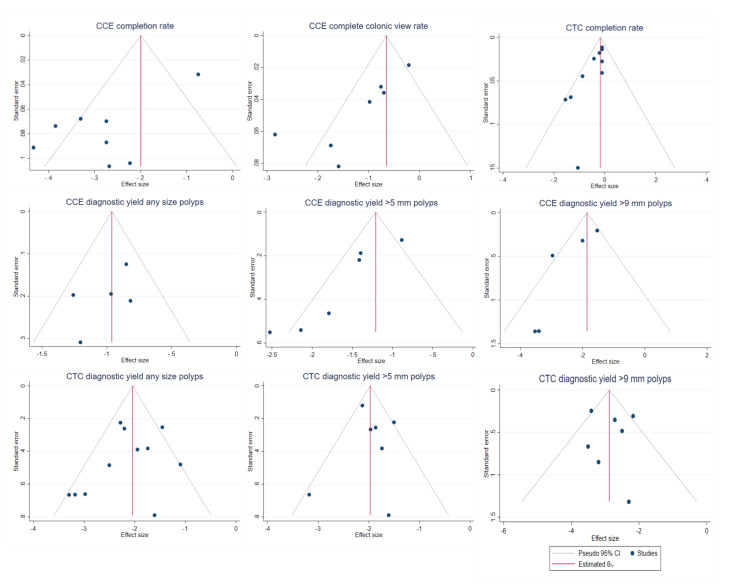
Funnel plots from Egger’s tests visualizing the symmetry of the effect sizes within each sub-analysis from the meta-analysis on colon capsule endoscopy vs. CT colonography following incomplete colonoscopy.

Egger’s test was performed for each subgroup testing for small study effects and publication bias. Egger’s test was significant (*p* < 0.05) for the CCE completion rate, CCE complete colonic view rate and CTC completion rate. For the CCE diagnostic yield, Egger’s test was not significant for polyps of any size but was for >5-mm and >9-mm polyps. For the CTC diagnostic yield, Egger’s test was not significant for any of the polyp size groups. Funnel plots are included in Appendix B, Figure A1. Funnel plots from significant Egger’s tests all seem to have an overweight of studies under the estimated effect size (skewed to the left). This may be due to publication bias, small study effects or differing study characteristics. Publication bias, i.e., studies reporting large effect sizes, are less likely to be published. Small study effects, i.e., that unusually high or low effect sizes are observed in smaller study samples due to chance, which then skews the symmetry of the funnel plots. Differences between study characteristics, such as methods or populations, i.e., study effect sizes deviating from the rest, have included populations either more or less responsive to the investigation or have conducted the investigation or analyses differently than their peers.

## 4. Discussion

The diagnostic yield of CCE for polyps of any size was almost fourfold compared to CTC. For polyps >5 mm and >9 mm, we observed approximately twofold nonsignificant differences in the diagnostic yields. This indicates that CCE is superior to CTC, especially in detecting small polyps. There was insufficient empirical evidence for evaluating the sensitivity. The completion rate of CTC (98% CI 95% 96–100%) was superior to that of CCE (76% CI 95% 68–84%), but the complete colonic view rate of CCE was 90% (CI 95% 83–95%). As described in the introduction, the complete colonic view rate seems appropriate for comparison with the CTC completion rate, as it completes the diagnostics. It may be acceptable for CCE to have a slightly lower complete colonic view rate than a CTC completion rate if more patients have polyps detected.

Some limitations to the studies in our analyses were present. The CCE studies reported completion rates, but not all CTC studies did. If the completion rate or poor investigations were not described, the study could not be included for the completion rate analysis. If CTC studies with nonreported completion rates were actually due to all investigations being complete, this may have resulted in an underestimation from our analysis. Since the pooled estimate was 98%, it could only be underestimated by a maximum of two percentage points. Further, information on the colon distension, residue levels in the colon and fecal tagging were not extracted or evaluated, as we believe them to be part of the completion assessments of each investigation. The quality of the CCEs and CTCs may therefore vary between or within studies. As the health care professionals conducting the investigations have regarded them sufficient, they should be included in the analyses to ensure that our estimates represent reality. Only studies in which the authors clearly stated the number of complete or incomplete investigations were included.

Colonoscopy must be considered the reference standard for colonic polyp detection. No study systematically performed colonoscopy after negative CCE or CTC investigations. This made it impossible to determine whether the higher diagnostic yield of CCE was subject to bias by either a large proportion of false-positive polyps from CCE, a large proportion of false-negative polyps from CTC or both. Colonoscopy is not a perfect diagnostic tool for colonic polyps, and significant miss rates of 17–25% have been reported [40,41,42], with a pooled estimate of 22% [43]. Therefore, when colonoscopy does not confirm polyps, they are not necessarily false positives. Although it was not possible to calculate the sensitivities, two studies reported the relative sensitivity of CCE compared to CTC as 2.0 and 2.7 for polyps >5 mm and as 1.7 and 1.9 for polyps >9 mm [4,5].

The diagnostic yield may differ between patient groups, i.e., screening, surveillance and symptomatic patients, which could result in bias if one group were over- or underrepresented in either CTC or CCE studies. This seems unlikely to be the case, as all three subgroups were heavily represented in the included studies of each procedure.

Two CCE studies applied the first-generation capsule, whereas the remaining applied the second (Table 2). Previously, the second-generation capsule proved more sensitive to polyps than the first-generation capsule [44]. This may have contributed to the heterogeneity observed. When the patterns of completion rates and diagnostic yields (Figure 2, Figure 3, Figure 4 and Figure 5) are considered, it becomes evident that only for polyps >9 mm does a first-generation PillCam study seem to perform worse than the second. CCEs have not been conducted for as many years as CTC, resulting in a much wider timespan of published articles regarding CTC. However, limiting the analyses to studies published after 2009 had little to no effect on the pooled estimates.

Our methods also hold some limitations. Heterogeneities were present in most of the analyses, indicating the presence of variances between the studies. Egger’s tests were significant in several analyses, indicating the presence of publication bias and/or small study effects. The results should therefore be interpreted with these limitations in mind. No quality assessment, other than fulfilling the inclusion criteria, was made. We extracted raw data from the studies and calculated the completion rate and diagnostic yield. If a study used an inappropriate way of analyzing the data, it would therefore not affect our results, as we systematically treated the raw data equally. The possible inclusion of low-quality studies could affect the estimates and lead to heterogeneity, but lower quality studies would have to be underestimating the effects of CTC and overestimating the effects of CCE to explain the observed differences, and the Eggers plots do not suggest this to be true. It is possible that reaching out to authors of excluded papers could have led to the inclusion of more studies, although with a risk of bias if certain authors were more likely to respond or share data. This was omitted, since it was our desire to have a nondifferential approach to all articles identified. Moreover, our study approach was very systematic, with clearly defined inclusion criteria, and no study was included unless we were certain that we could identify and extract the data needed for our analyses. Expanding the literature search to additional scientific research databases or systematically adding keywords from studies identified through snowballing might also have increased the number of included studies, but since thorough snowballing was conducted and further repeated in the newly identified studies, we believe that the search reached the point of exhaustion.

CTC may be more reliable for larger polyps than smaller polyps [9,45]. This was also confirmed by our meta-analysis, as the difference in the diagnostic yield between CCE and CTC was reduced when limiting the analyses to polyps >5 mm and >9 mm. CTC and CCE have the drawback of being diagnostic investigations, as a positive test result would have to be followed by therapeutic procedures. IC, as well as standard colonoscopy, can be very painful, and sometimes, pain is even the reason for incompletion [5]. Therefore, patients may prefer to have a confirmed finding prior to further colonoscopy attempts. Patient preference should also be considered when comparing CTC and CCE, as bowel preparation regimens and the nature of the investigations differ substantially. Implementing CCE would require trained CCE readers. Training would therefore need to be incorporated alongside training in traditional endoscopy. CTC usually includes a simpler bowel preparation; is able to identify extracolonic findings not available from CCE (e.g., hepatic and renal cysts, lung nodules, adrenal adenomas and extracolonic malignancy); is less costly; is more widely available compared to CCE and does not include the ingestion of equipment. CCE seems preferred by patients [15], spares the patient unnecessary radiation and is possible to conduct in the same unit (without referral to another clinic in the same or another hospital) or even in the privacy of their own home.

## 5. Conclusions

This meta-analysis of CCE vs. CTC following IC indicates that a complete CCE after IC is not inferior to CTC in detecting polyps. The completion rate of CTC was higher than that of CCE. However, when comparing the complete colonic view rate of CCE to the CTC completion rate, the difference was reduced. The diagnostic yield for any size polyps was almost fourfold in CCE compared to CTC and twofold, but statistically insignificant, in medium-large and large-sized polyps. The increased diagnostic yield of CCE could outweigh its slightly lower complete colonic view rate compared to the superior CTC completion rate. Hence, CCE following IC appears feasible for an introduction to clinical practice. Therefore, randomized studies investigating CCE and/or CTC following incomplete colonoscopy with a reference standard for the entire population, enabling estimates for sensitivity and specificity, are needed. The empirical evidence is still scarce, especially for CCE, and the analyses should be repeated when more studies are available.

## Figures and Tables

**Table 1:** Data extracted for statistical analyses. IC: incomplete colonoscopy, CCE: colon capsule endoscopy and CTC: computed tomography colonography.

Number	Description
I	Number of individuals/investigations included in the study
II	Number of individuals with an IC previous to CCE/CTC
III	Individuals with polyp(s) (any size polyp, >5 mm polyp and >9 mm polyp)
IV	Number of complete investigations
V	Number of investigations that combined with IC achieved complete colonic view
VI	True positive individuals (any size polyp, >5 mm polyp and >9 mm polyp)
VII	True negative individuals (any size polyp, >5 mm polyp and >9 mm polyp)
VIII	False positive individuals (any size polyp, >5 mm polyp and >9 mm polyp)
IX	False negative individuals (any size polyp, >5 mm polyp and >9 mm polyp)

**Table 2:** Overview of 26 studies included for meta-analysis on colon capsule endoscopy vs. CT colonography after incomplete colonoscopy.

Publication	Study Type	Visualization Instruments (PillCam/CT Scanner)	Sample Size (Complete Sample Size)
CCE studies			
Hussey—2018 [10]	Prospective clinical	PillCam COLON 2	50 (50)
Alarcon-Fernandez—2013 [35]	Prospective clinical	PillCam COLON 1	34 (34)
Baltes—2019 [11]	Prospective clinical	PillCam COLON 2	74 (74)
Nogales—2017 [2]	Prospective clinical	PillCam COLON 2	96 (96)
Triantafyllou—2014 [3]	Prospective clinical	PillCam COLON 1	75 (75)
Spada—2015 [4]	Prospective clinical	PillCam COLON 2	97 (97)
Deding—2020 [5]	Prospective clinical	PillCam COLON 2	97 (97)
Negreanu—2013 [39]	Prospective clinical	PillCam COLON 2	30 (70)
CTC studies			
Iafrate—2008 [37]	Prospective clinical	General electric (GE): 64-volume computed tomography scanner (VCT)	136 (136)
Britton—2001 [32]	Prospective clinical	Phillips: Tomoscan A.V. helical CT system	23 (50)
Bollart—2015 [1]	Retrospective register	NA	59 (297)
Maggialetti—2016 [22]	Prospective clinical	Toshiba: 320-row CT scanner	61 (61)
Lai—2009 [21]	Retrospective chart review	GE: multi-detector computed tomography scanner (MDCT)	196 (764)
Neerincx—2010 [24]	Prospective cohort	NA	12 (511)
Ratnasingham—2017 [27]	Retrospective register	GE: 64 slice scanner	81 (200)
Luo—2002 [34]	NA	HiSpeed advantage spiral CT scanner	60 (60)
Copel—2007 [36]	Retrospective chart review	GE, MDCT: GE Lightspeed QX/i four-section or GE Lightspeed ultra eight-section	546 (546)
Gryspeerdt—2005 [33]	Retrospective and prospective clinical	GE: HiSpeed CT/i scanner	49 (49)
Macari—1999 [31]	Prospective clinical	GE: HiSpeed advantage scanner	10 (20)
Morrin—1999 [23]	Prospective clinical	GE: CT scanner or somatom plus 4 spiral CT scanner	40 (40)
Neri—2002 [30]	Prospective clinical	GE: single detector row spiral CT scanner	34 (54)
Neri—2010 [25]	Prospective clinical	GE: 4 or 64 row CT scanner	72 (72)
Pullens—2013 [26]	Retrospective chart review	Philips: 16- or 64-slice MDCT	136 (136)
Salamone—2011 [28]	Prospective clinical	16-detector CT scanner or dual source CT scanner	68 (68)
Sali—2008 [29]	Prospective cohort	Siemens: 16-MDCT scanner (Sensation 16)	42 (42)
Spada—2015 [4]	Prospective clinical	GE: 64-volume CT (VCT) scanner	100 (100)
Deding—2020 [5]	Prospective clinical	Siemens: Somatom Definition Edge 64-slice CT scanner	97 (97)
O’Shea—2020 [38]	Retrospective chart review	NA	245 (245)

Sample size: sample included for meta-analysis, CCE:colon capsule endoscopy, CTC: computed tomography and NA: not available.

## Appendix A

### Search Strings:

Web of Science: 524 Hits (661 before Limiting to Articles)

ALL = (“Capsule endoscop*” OR “Camera endoscop*” OR WCE* OR PillCam* OR “Pill cam*” OR “camera pill*” OR “capsule camera*” OR “wireless camera*” OR “Endoscopic capsule*” OR “endoscopy capsule*” OR CCE* OR Colonograph* OR CTC*) AND ALL = (Colonoscop*) AND ALL = (Cohort OR “Randomized controlled trial*” OR RCT* OR case-control OR “case control” OR paired OR prospective)

PubMed: 739 Hits (754 before Limiting to Journal Articles)

(“Capsule endoscop*” OR “Camera endoscop*” OR WCE OR PillCam* OR “Pill cam*” OR “camera pill*” OR “capsule camera*” OR “wireless camera*” OR “Endoscopic capsule*” OR “endoscopy capsule*” OR CCE OR “Capsule Endoscopy” (mh) OR “Capsule Endoscopes” (mh) OR Colonograph* OR CTC OR “Colonography, Computed Tomographic” (mh)) AND (Colonoscop* OR Colonoscopy (mh)) AND (cohort OR “Randomized controlled trial*” OR RCT OR case-control OR “case control” OR paired OR prospective OR “Cohort Studies” (mh) OR “Randomized Controlled Trial” (mh) OR “Case-Control Studies” (mh) OR “Matched-Pair Analysis” (mh) OR “Prospective Studies” (mh))

Embase: 551 Hits (1122 before Limiting to Articles)

((“Capsule endoscop*” or “Camera endoscop*” or WCE* or PillCam* or “Pill cam*” or “camera pill*” or “capsule camera*” or “wireless camera*” or “endoscopic capsule*” or “endoscopy capsule*” or CCE* or capsule endoscope/ or capsule endoscopy/ or colonograph* or CTC* or computed tomographic colonography/) and (Colonoscop* or colonoscopy/) and (cohort or “Randomized controlled trial*” or RCT* or case-control or “case control” or paired or prospective or cohort analysis/ or randomized controlled trial/ or case control study/ or prospective study/))

**Table A1:** Search strategy to identify the relevant studies on colon capsule endoscopy vs. CT colonography following incomplete colonoscopy.

	AND			
	Investigation		Indication	Study Design
**OR**	Capsule camera *, Wireless camera *, Endoscopic capsule*, Endoscopy capsule *, CCE *, Capsule endoscop *, Camera endoscop *, WCE *, PillCam *, PillCam *, Camera pill *, Capsule Endoscopy (mh), Capsule Endoscopes (mh), Capsule endoscope/, Capsule endoscopy/,	Colonograph *, CTC *, Colonography, Computed Tomographic (mh), Computed tomographic colonography/,	Colonoscop *, Colonoscopy (mh), Colonoscopy/,	Cohort, Randomized controlled trial *, RCT *, Case-control, Case control, Paired, Prospective, Cohort Studies (mh), Randomized Controlled Trial (mh), Case-Control Studies (mh), Matched-Pair Analysis (mh), Prospective Studies (mh), Cohort analysis/, Randomized controlled trial/, Case control study/, Prospective study/

*—truncation identifying all terms regardless of term form. (mh)—Medical Subject Heading (MeSH) used in PubMed/—Subject Heading used in Embase.
